# From Genes to Lives: Integrating the Complexities of Primary Ovarian Insufficiency

**DOI:** 10.3390/ijms27031353

**Published:** 2026-01-29

**Authors:** Rand Abujaber, Charnae Henry-Smith, Sudha Sharma

**Affiliations:** 1 Department of Biochemistry and Molecular Biology, College of Medicine, Howard University, 520 W Street, NW, Washington, DC 20059, USA; rand.abujaber@bison.howard.edu; 2 Department of Biology, College of Arts and Sciences, Howard University, 415 College Street, NW, Washington, DC 20059, USA; 3 National Human Genome Center, College of Medicine, Howard University, 520 W Street, NW, Washington, DC 20059, USA

**Keywords:** primary ovarian insufficiency (POI), etiology, genetic and epigenetic mechanisms, reproductive aging, psychosocial impact, health disparities

## Abstract

Primary ovarian insufficiency (POI) affects up to 3% of reproductive-aged women and is a critical yet underrecognized contributor to infertility and systemic accelerated aging. While most cases remain idiopathic, advances in genomics increasingly reveal a genetic basis, implicating pathways that govern DNA repair, meiosis, chromosomal stability, and folliculogenesis. This review synthesizes the multifactorial etiology of POI, integrating genetic contributions with emerging evidence on epigenetic dysregulation, mitochondrial dysfunction, and environmental influences such as toxins and lifestyle factors. These mechanisms converge on core cellular processes, driving premature follicular depletion and shortening reproductive lifespan. We also highlight racial and ethnic disparities in POI prevalence and research representation, alongside the profound psychosocial burden experienced by affected individuals. Addressing these challenges through integrative strategies that unite mechanistic insight with equity is essential, not only for improving POI care but also for advancing precision approaches to ovarian aging and safeguarding reproductive health across the lifespan.

## 1. Introduction

Aging is a gradual, inevitable process that affects all organ systems, including the reproductive axis in women. This physiological decline in follicle number and quality culminates in menopause, a universal midlife milestone defined by the permanent cessation of menstrual cycles, typically occurring around age 50, with an average onset at 51 [1]. In contrast, Primary Ovarian Insufficiency (POI) represents a pathological, premature transition, often before age 40, leading to early ovarian failure and hormonal disruption. POI affects approximately 1 in 100 women by age 40 and 1 in 1000 by age 30 [2,3,4,5,6], with consequences for fertility and overall health. Seminal work by Ruth et al. (2021) has highlighted that the genetic architecture of reproductive aging often overlaps with that of POI, suggesting a shared biological continuum between natural menopause and pathological ovarian failure [7].

POI, also known as premature ovarian failure (POF), presents with a range of clinical phenotypes often characterized by the absence or disruption of menstruation (primary or secondary amenorrhea), increased gonadotropin levels, and low estradiol levels [3,8,9]. Primary amenorrhea is defined as the failure to begin menstruation by the age of 14, along with the absence of secondary sexual characteristics, or the failure to menstruate by age 16, regardless of sexual development [10]. Secondary amenorrhea refers to the cessation of menstruation for more than 3 months in a woman with previously regular periods or for more than 6 months in a woman with irregular cycles [11]. Additional symptoms of POI include fatigue, anxiety or depression, hot flashes, sleep disturbances, and decreased libido [12]. Early ovarian failure often leads to infertility, though spontaneous pregnancy has been reported to occur in 5–10% of cases [13,14].

Diagnosis of POI is often delayed, as symptoms are frequently mistaken for other diseases or overlooked [11,15,16]. Diagnostic criteria include menstrual irregularities before age 40 and abnormal hormone levels. According to international consensus, including guidelines from the European Society of Human Reproduction and Embryology (ESHRE), diagnosis is supported by follicle-stimulating hormone (FSH) levels exceeding 25 IU/L, which is below the previously suggested threshold of >40 IU/L, on two occasions at least 4 weeks apart [17,18]. Further tests may include karyotyping and *FMR1* gene testing, screening for autoimmune conditions, physical exams to evaluate the presence and development of secondary sex characteristics, and pelvic ultrasounds to assess ovarian health, follicle counts, and endometrial thickness. Given the strong association with autoimmune disease, screening for thyroid (TSH, TPO-Ab) and adrenal (21-hydroxylase Ab) autoimmunity is also recommended [3,17,18,19,20].

Management of POI primarily centers on hormone replacement therapy (HRT) and fertility interventions [17,21]. HRT typically involves the administration of estrogen, progesterone, and androgens to alleviate hypoestrogenic symptoms and reduce long-term risks such as osteoporosis and cardiovascular disease [3,17,21]. Menopausal hormone therapy may help mitigate accelerated biological aging associated with POI, underscoring the systemic protective benefits of hormone therapy [22,23]. However, it does not restore ovarian function. For patients seeking pregnancy, oocyte donation remains the most effective option, while cryopreservation offers fertility preservation for those at risk of POI [24]. Promising approaches, such as stem cell therapies, in vitro activation, mitochondrial activation, and platelet-rich plasma infusion, are being explored for their potential to rejuvenate ovarian tissue and improve reproductive outcomes [3,24,25,26,27,28,29]. These innovations represent a shift toward restoring ovarian function rather than solely managing its loss.

This review synthesizes the multifactorial etiology of POI, integrating known genetic contributions with emerging evidence on epigenetic dysregulation, mitochondrial dysfunction, and environmental influences. We prioritized studies identifying causative germline variants, validated animal models, and recent large-scale cohorts to provide an up-to-date narrative on the biological and societal complexities of POI.

## 2. Biological Context: Oogenesis and Physiological Ovarian Decline

Understanding POI requires a foundational grasp of the ovary’s developmental and aging trajectory [1,30,31,32,33,34]. Oogenesis begins in utero when primordial germ cells (PGCs), specified in the epiblast, migrate to the gonadal ridges around embryonic weeks 5–6. This migration is accompanied by extensive proliferation. Upon reaching the gonads, PGCs differentiate into oogonia, which enter meiosis I and arrest at the diplotene stage of prophase I, a state they may remain in for decades. Each arrested oocyte becomes encapsulated by pre-granulosa cells, forming a primordial follicle. This marks the onset of folliculogenesis, the process by which follicles mature through primary, secondary, and antral stages. This critical developmental window is orchestrated by a precise interplay of genetic networks and epigenetic programming, establishing the oocyte’s competency and long-term survival [32,35].

The ovarian reserve peaks at approximately 6–7 million oocytes around 20 weeks of gestation, followed by a dramatic wave of apoptosis, leaving about 1 million oocytes at birth. By puberty, only 300,000 remain. This decline continues steadily; by age 30, roughly 100,000 oocytes persist, and by age 40, the number drops to 10,000. Menopause typically ensues when fewer than 1000 follicles remain [36].

This age-related attrition reflects a qualitative deterioration driven by molecular changes such as telomere shortening and reduced expression of DNA repair genes and cohesin loss, all of which impair chromosomal segregation [37,38]. These changes compromise oocyte integrity and mirror the molecular signatures observed in POI [32,36], suggesting that POI represents an accelerated form of physiological ovarian aging.

Throughout oogenesis, quality control mechanisms, including DNA damage checkpoints (ATM/ATR) and apoptotic pathways, maintain genomic fidelity and eliminate damaged or aneuploid oocytes [32,35,36]. The tightly regulated progression from embryonic germ cell specification to age-related follicular depletion underscores ovarian aging as a continuous physiological process [37] and supports the view that POI is a premature manifestation rather than a distinct pathology.

Consistent with this model, a 2025 study by Zhou et al., analyzing over 229,000 women from the UK Biobank and NHANES, provided the first large-scale evidence linking POI to accelerated biological aging [22].

## 3. Non-Genetic and Environmental Etiologies

**Figure 1:**
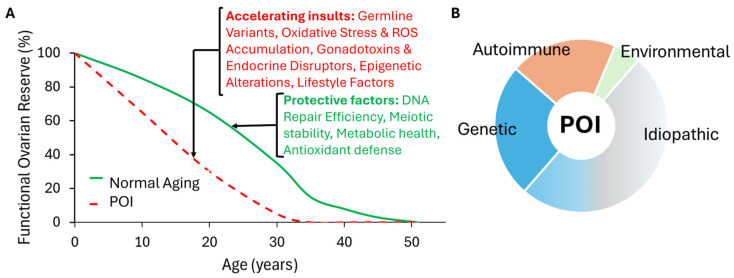
**Pathogenesis and Etiology of Primary Ovarian Insufficiency**. (**A**) **Accelerated Ovarian Aging Trajectory.** Physiological ovarian reserve (green) supports reproductive function until natural menopause (~51 years). Pathogenic insults, including germline variants and altered epigenetics, accelerate follicular atresia, shifting the trajectory (red dashed line) toward premature ovarian exhaustion before age 40, resulting in POI. (**B**) **Etiological Landscape.** Genetic defects account for ~20–25% of POI cases, autoimmune factors for ~20–30%, and environmental/iatrogenic causes for ~5–10%. The idiopathic fraction (~40–50%) is depicted as a gradient interface to reflect ongoing reclassification of cases as genetic, polygenic, or epigenetic with emerging genomic data.

POI is a multifactorial condition with diverse etiologies [3,15,24,28,39]. While genetic causes are increasingly identified, a majority of POI cases remain idiopathic or are attributed to environmental, iatrogenic, or metabolic insults (Figure 1). Understanding these non-genetic contributors is essential for identifying modifiable risk factors.

### 3.1. Environmental, Iatrogenic, and Lifestyle Factors

Non-genetic causes of POI include iatrogenic factors, such as ovarian surgeries, chemotherapy, and radiation [40]. These gonadotoxic treatments directly impair ovarian reserve; for example, alkylating agents like cyclophosphamide work by inducing double-strand breaks (DSBs) and apoptosis in oocytes [41].

Environmental exposures, including cigarette smoke [6], pesticides [42], and endocrine disruptors like bisphenol A (BPA) [43], have also been implicated in POI and accelerating ovarian aging. These agents often act by promoting oxidative stress pathways, impairing DNA repair gene expression, and disrupting granulosa cell function, which accelerates follicular atresia [40,41]. A meta-analysis of 38 studies identified modifiable risk factors such as chemical exposure, smoking, sleep deprivation, and psychological stress [39]. Occupational exposures, including hair dye chemicals, were associated with a fivefold increase in POI risk, underscoring the need for environmental health surveillance in reproductive-age women [39].

Dietary and lifestyle influences play a significant, albeit often underappreciated, role in ovarian health [44]. Obesity and low BMI exacerbate oxidative stress and inflammation, creating a hostile microenvironment for follicular development, while diets rich in omega-3 fatty acids and antioxidants (melatonin, CoQ10) may offer protective effects by neutralizing reactive oxygen species (ROS) and supporting mitochondrial function [44]. Emerging evidence also implicates gut microbiota dysbiosis in hormonal disruption, ovarian aging, and POI [45]. Collectively, these factors suggest that lifestyle modifications could serve as an adjuvant strategy for preserving ovarian function.

Metabolic disorders and autoimmune diseases (e.g., Addison’s disease, thyroiditis) contribute to POI through systemic inflammation and immune-mediated ovarian damage [3,15,22,46,47,48,49]. Autoimmune etiologies alone account for approximately 20–30% of cases, highlighting the overlap between systemic immunity and reproductive longevity [50].

### 3.2. Mitochondrial Dysfunction

Mitochondrial integrity is central to oocyte quality and survival. Many environmental and metabolic insults compromise mitochondrial integrity via excessive ROS production [23,49]. Mitochondrial dysfunction amplifies ROS generation, creating a vicious cycle that impairs ATP synthesis, steroidogenesis, and oocyte quality [51]. Recent evidence links mitochondrial biogenesis defects, fusion-fission imbalance, and mitochondrial DNA mutations to POI pathogenesis, thereby disrupting energy metabolism and calcium homeostasis, which are critical for follicular development [51,52,53]. Thus, mitochondrial dysfunction is emerging as a central mechanism in ovarian aging and POI, bridging the gap between environmental insults and cellular senescence [3,54,55].

## 4. Genetic Basis of POI

### 4.1. General Principles of POI Genetics

Genetic factors contribute significantly to the etiology of POI, accounting for approximately 25% of cases, with both X-linked and autosomal genes implicated [2,3,28,56,57,58]. These genes regulate essential molecular processes, including DNA repair, meiosis, folliculogenesis, and oocyte maturation, all of which are critical for maintaining ovarian function throughout the reproductive lifespan. The prolonged arrest of oocytes in prophase I makes them especially reliant on these pathways [34], highlighting the vulnerability of the ovarian reserve to genetic disruption. Importantly, the variants associated with POI are predominantly constitutional (germline) mutations, present in all somatic and germ cells, rather than somatic mutations typical of neoplastic processes.

The genetic architecture of POI is heterogeneous, ranging from macroscopic chromosomal abnormalities to single-nucleotide variants (SNVs) and copy number variations (CNVs). Chromosomal defects [56,59], such as Turner syndrome (monosomy X) [60] and X-autosome translocations [61], have historically accounted for a significant portion (approximately 10–13%) of diagnosed POI cases [3,39,62], primarily by disrupting the “critical region” on the X chromosome (Xq13-q26) necessary for ovarian maintenance.

### 4.2. Known Genetic Contributors to POI

Advances in high-throughput sequencing have revealed a rapidly expanding set of autosomal dominant, autosomal recessive, and X-linked genes implicated in POI, highlighting an increasingly complex genetic landscape. Hundreds of candidates continue to emerge as sequencing cohorts expand. In this review, we focus on major functional pathways and representative gene defects, directing readers to comprehensive catalogs in recent genetic reviews such as Nie et al. [63]. Below, we summarize key genetic contributors organized by biological function, with Table 1 providing a non-exhaustive overview of genes implicated in POI.

**a. DNA Repair and Genome Integrity:** Oocytes arrested in prophase I are particularly reliant on robust DNA repair mechanisms to maintain genomic fidelity over decades [64]. Mutations in genes central to homologous recombination (HR), the primary pathway for repairing meiotic DSBs, are strongly associated with POI [65,66]. Key genes in this pathway include *BRCA2*, where compound heterozygous mutations impair HR, leading to oocyte apoptosis and gonadal dysgenesis, and *FANCA* and *FANCM*, which are critical for DNA damage repair and genomic stability during oogenesis [67,68,69,70,71]. Mutations in *DMC1* disrupt the repair of meiotic DSBs [72,73], while defects in mismatch repair (MMR) genes like *MSH5* (e.g., p.D486Y mutation) and *MSH4* (e.g., c.2355+1G>A mutation) impair meiotic crossover formation, leading to meiotic arrest at prophase I and oocyte depletion [74,75]. Genes involved in non-homologous end joining (NHEJ) also contribute to genomic instability when mutated [66,76]. The transcription factor TP63 also plays a pivotal role in safeguarding oocyte genome integrity; gain-of-function mutations in *TP63* induce oocyte apoptosis and accelerate oocyte depletion [77].

**b. Meiotic Regulation and Chromosomal Stability:** Proper chromosomal segregation during meiosis depends on the cohesin complex and synaptonemal complex formation [78]. Mutations in genes encoding these components, such as *STAG3* (sister chromatid cohesion) and *SYCE1* (synaptonemal complex), disrupt chromosome pairing and are linked to ovarian atrophy and primary amenorrhea [79,80,81]. Similarly, mutations in *HFM1* and *MEIOB*, which impair single-strand DNA binding during DSB repair, result in incomplete meiotic recombination and oocyte loss [82,83,84]. Other meiotic regulators like *SHOC1*, *KASH5*, and *MCMDC2* have also been implicated in disrupting meiotic efficiency [57,65,72].

**c. Folliculogenesis, Oocyte Maturation, and Signaling:** A large network of genes regulates the development of the follicle and oocyte, including many previously classified as “non-syndromic” causes of POI [85,86]. Early establishment and maintenance of the primordial follicle pool depend on transcription factors essential for gonadal development and follicle specification, such as *FIGLA*, *NOBOX*, *LHX8*, and *NR5A1* [87,88,89,90,91,92,93,94]. Disruption of these factors compromises folliculogenesis at its earliest stages.

Follicle activation and survival are further regulated by signaling pathways and cell-intrinsic factors. Mutations in *GPR3*, *WT1*, *FOXO3α*, and *SOHLH1* lead to premature follicular activation, apoptosis, and impaired granulosa cell development [95,96,97,98]. Components of the Wnt signaling pathway, including *LRP5* and *LGR4*, are similarly critical for ovarian development, with pathogenic variants associated not only with POI but also with systemic phenotypes such as osteoporosis [99].

Gonadotropin responsiveness represents a key checkpoint in follicle recruitment and growth. *FSHR* mutations (e.g., p.A189V) are a well-known cause of follicular recruitment failure and often exhibit ethnic associations [100,101,102,103,104]. Proper follicle growth also depends on bidirectional communication between the oocyte and surrounding granulosa cells, mediated by oocyte-derived growth factors such as GDF9 and the X-linked BMP15, which are indispensable for granulosa cell function and coordination [105,106,107,108,109,110].

As follicles progress, oocyte maturation and specialization rely on genes responsible for structural integrity and germ cell identity. *ZAR1*, *ZP3* (critical for zona pellucida formation), and *PRDM1* (a regulator of primordial germ cell development) are essential at this stage [57,111]. Germ cell survival further depends on transcriptional regulators such as *POF1B*, *DACH2*, *SOHLH2*, and *SALL4* [89,112]. Finally, underscoring the complexity of transcriptional control during meiosis, heterozygous loss-of-function variants in *MGA*, a key regulator of meiotic gene expression, have been linked to reduced ovarian reserve [113].

**d. Syndromic Genes:** In these cases, POI is one feature of a multi-system disorder. Key examples include *FOXL2* (linked to Blepharophimosis-Ptosis-Epicanthus Inversus Syndrome (BPES) Type II), *GALT* (associated with galactosemia), *AIRE* (part of autoimmune polyglandular syndrome), *POLG* (linked to mitochondrial repair deficits), and *PMM2* (disrupting glycosylation) [114,115,116,117,118,119,120]. The *FMR1* premutation (55-200 CGG repeats), associated with Fragile X syndrome, is the most common single-gene cause and is found in 0.8–7.5% of sporadic cases and up to 13% of familial POI [117,121,122], highlighting its relevance in genetic screening.

**e. New Risk and Candidate Genes:** Large-scale sequencing efforts continue to identify novel POI candidate genes [57,58,123]. These include genes involved in fundamental cellular processes such as protein regulation (*USP36*, *NPM2*, *VCP*), mRNA processing (*WDR33*, *EIF4ENIF1*), germline integrity (*PIWIL3*), and transcriptional regulation (*POLR2C*, *POLR2E*, *POLR2H*).

**f. Androgen Metabolism:** Beyond the well-characterized DNA repair and meiotic genes, androgen metabolism pathways also contribute to POI risk. Polymorphisms in genes regulating androgen metabolism, such as *SULT2A1*, *CYP19A1*, *SRD5A2*, and *AR*, that influence DHEA conversion to testosterone, a process essential for early folliculogenesis [124]. Extended CAG repeats in the *AR* gene reduce receptor activity and are associated with diminished ovarian reserve [125]. These findings highlight the importance of integrating hormonal and metabolic profiling into genetic screening strategies for POI.

**Table 1:** Key Genes Implicated in Primary Ovarian Insufficiency.

Category	Gene	Function/Pathway	Key Findings and Phenotypes
a. DNA Repair and Genome Integrity	*DMC1*	Meiotic DSB repair, HR	Missense mutations associated with POI [73]. One study found over 50% of its sub-Saharan African cohort had a variant, suggesting a potential biomarker in Black populations [126].
	*BRCA2*	HR, RAD51 recruitment	Compound heterozygous mutations caused primary amenorrhea, short stature, and gonad dysgenesis [69].
	*FANCA*	FA pathway, DNA repair, meiosis	Mutations implicated in sporadic POI [67]. Heterozygous mice had reduced fertility and smaller ovaries [71].
	*FANCM*	DNA damage repair (HR)	Homozygous mutation identified in familial POI cases. Defective repair can lead to accelerated follicle depletion [127].
	*MSH5*	Mismatch repair (MMR), HR	Homozygous missense mutations (p.D487Y) in a Han Chinese family led to amenorrhea and atrophic ovaries. The mouse model showed meiosis arrest [74].
	*MSH4*	Meiotic recombination	Rare cause of POI. A specific mutation (c.2355+1G>A) may alter the protein, preventing it from binding MSH5 [68,75].
	*CSB-PGBD3*	Transcription-coupled DNA repair	Mutations associated with POI impaired DNA damage repair and affected ovarian development [128].
	*TP63*	Transcription factor, safeguards germline genome integrity	Heterozygous variants (0.78% of POI cases) disrupt autoinhibition, leading to activation of proapoptotic genes and oocyte depletion [77].
b. Meiotic Regulation	*STAG3*	Cohesin complex, sister chromatid pairing	Variants associated with primary amenorrhea underdeveloped breasts [81,129]. Deficient mice had fibrotic ovaries with no follicles [81].
	*SYCE1*	Synaptonemal complex component	Deficient mice are infertile [79]. A homozygous missense mutation was found in affected sisters in a Middle Eastern family [80].
	*HFM1*	ATP-dependent DNA helicase, HR	Variants observed in 2% of POI patients [130]. Missense mutations linked to splicing abnormalities affecting oocyte development [82,130,131].
	*MEIOB*	Single-strand DNA binding during meiotic DSB repair	Homozygous variant led to protein truncation [84]. An estimated 1 in 11,000 women with POI have a variant [84]. Knockout mice lacked oocytes [132].
	*MCMDC2*	Homolog alignment, crossover formation	Variants disrupt functional domains and reduce HR efficiency, contributing to POI [57].
	*SPIDR*	DNA damage repair (interacts with BLM)	A novel heterozygous deletion (p. IIe616_Asp618del) potentially disrupts DNA repair [57,133].
	*SLX4*, *SHOC1*, *RFWD3*, *NUP43*, *MEIOSIN*, *KASH5*, *CPEB1*	Meiotic initiation, homologous pairing, and HR	Animal models show infertility, ovarian atrophy, and meiotic arrest [57,65,112]. *SHOC1* and *KASH5* variants also linked to male non-obstructive azoospermia [134,135].
c. Folliculogenesis and Signaling	*FSHR*	FSH signaling, follicle growth	First gene linked to non-syndromic POI [103]. rs6166 polymorphism linked to POI in Asian populations [104]; p.A189V mutation in Finnish cohort [103].
	*GDF9*	Follicular development, granulosa cell maturation	Heterozygous transversion (S186Y) found in a Caucasian woman with atrophic ovaries [107]. A homozygous truncating variant (c.604C>T; p.(Gln202Ter) also reported [110].
	*BMP15*	Oocyte-specific, granulosa cell growth	Variants reduce synergy with GDF9, leading to poor oocyte quality [105,107]. Homozygous mutations linked to folliculogenesis failure [136].
	*FIGLA*	Germ-cell-specific transcription factor	Heterozygous deletions linked to smaller uteruses and atrophic ovaries [87,88].
	*NOBOX*	Transcription factor, ovarian development	First autosomal gene linked to POI [91]; 42% of patients with a *NOBOX* variant were of sub-Saharan African descent [126].
	*LHX8*	Germ-cell-specific transcription factor	Deficiency in mice disrupted oocyte-specific gene expression [92]. A rare variant (p.Ala444Thr) found in POI patient [126].
	*NR5A1*	Transcriptional regulator, gonadal development	Mutations identified in POI patients [57,137] and other disorders of sex development [138].
	*FOXO3α*	Regulates ovarian oocytes, follicular development	Decreased mRNA expression seen in POI ovarian tissues [139]. Phosphorylation may reactivate primordial follicles [140].
	*ZAR1*	Folliculogenesis, oocyte maturation	Seven LoF variants identified, disrupting the conserved C-terminal ZNF domain [57]. *ZAR1* expression is negatively correlated with FSH levels in women with POI [141].
d. Syndromic Genes	*FMR1*	Encodes Fragile X Mental Retardation Protein (FMRP)	The first single-gene cause of premature ovarian failure [142,143,144]. Premutation (55–200 CGG repeats) associated with syndromic POI [121,145]. ~15% of premutation carriers develop POI [145].
	*FOXL2*	Transcription factor, ovarian development	First autosomal gene implicated in syndromic POI [146]. Mutations linked to Blepharophimosis/Ptosis/Epicanthus Inversus Syndrome (BPES) Type I, which presents with POI [147].
	*GALT*	Galactose processing	Mutations cause galactosemia; women frequently exhibit small ovaries and reduced follicle count GALT [115,148].
	*AIRE*	Autoimmune regulator, immune tolerance	Mutations linked to autoimmune polyglandular syndromes (APS) and hypogonadism [47,149].
	*POLG*	Mitochondrial DNA replication/repair	Mutations linked to mtDNA depletion and ovarian insufficiency in syndromic cases [118,150].
	*PMM2*	Glycosylation	Biallelic mutation can cause POI as part of *PMM2*-congenital disorder of glycosylation (*PMM2*-CDG) [119]; mutations reduce enzymatic activity, affecting oogenesis [112,120].
e. Androgen Metabolism	*AR*	Androgen Receptor	Extended CAG repeats reduce receptor activity and are associated with diminished ovarian reserve [125].
	*SULT2A1*	DHEA sulfation	Polymorphisms impair DHEA metabolism, common in African American women [124].
f. New Risk Genes (Various Functions)	*USP36*	Deubiquitinase, rRNA processing	A rare variant linked to POI and knockdown in Drosophila resulted in atrophic ovaries [123].
	*POLR2C*	RNA Polymerase II Subunit	Nonsense mutation in a family with dominant inheritance caused reduced mRNA levels, impairing germ cell proliferation [151].
	*WDR33*	mRNA processing (polyadenylation)	Highly expressed in oocytes; knockdown in Drosophila revealed atrophic ovaries [123].
	*PIWIL3*	Germline integrity, suppresses transposable elements	Expressed in oocytes; variants disrupting the PIWI domain linked to ovarian insufficiency [123].
	*NPM2*	Oocyte maturation, embryonic development	Variant linked to POI risk [123]. Highly expressed in oocytes; knockout models are infertile [152].
	*VCP*	ATPase, germinal vesicle breakdown	Missense mutations associated with POI and ovarian atrophy [123].
	*EIF4ENIF1*	Translational regulation	Dominant-inherited heterozygous *EIF4ENIF1* mutations in some POI cases [153,154]. Haploinsufficiency impairs fertility in the mouse model [155]

### 4.3. Epigenetic Regulation in POI

Epigenetic mechanisms, including DNA methylation, histone modifications, and non-coding RNAs, play a critical role in ovarian development, folliculogenesis, and reproductive aging [156]. Unlike genetic mutations, epigenetic changes alter gene expression without modifying the DNA sequence [157], offering dynamic and reversible control. In POI, these changes disrupt granulosa cell function, oocyte maturation, and follicular survival [158]. Specifically, epigenetic reprogramming during oogenesis involves genome-wide demethylation followed by de novo methylation to establish maternal imprints, a process essential for oocyte competence that is susceptible to disruption by environmental and genetic factors [159].

**a. DNA Methylation and Oocyte Senescence:** DNA methylation is the most extensively studied epigenetic mechanism in female reproductive aging [160]. Aberrant methylation patterns have been identified in both granulosa cells and oocytes from women with diminished ovarian reserve, a known precursor to POI [161]. These alterations disrupt gene regulatory networks essential for follicular development, hormone responsiveness, and oocyte competence.

Several genes critical to reproductive function are particularly sensitive to methylation changes [162,163,164]. For instance, proper methylation of *HOXA10* [165,166] and the progesterone receptor (*PGR*) [167,168] is required for endometrial receptivity and oocyte competency, and aberrant methylation at these loci has been significantly linked to implantation failure. Similarly, hypermethylation of the *ESR1* promoter can silence estrogen receptor expression, mimicking the hypoestrogenic state of POI [163,169]. Furthermore, altered methylation and expression of *MEG3*, a maternally expressed imprinted lncRNA, regulates oxidative stress and apoptosis in granulosa cells, directly impacting follicular reserve [158,170]. In addition, hypermethylation of CpG islands in genes such as *AMH* and *IGF2* suppresses hormone production and impairs oocyte development [164].

Notably, age-related methylation changes are increasingly recognized as determinants of oocyte quality and reproductive potential [162]. Experimental studies in mice demonstrate that aging alters *DNMT1* and *DNMT3a/b* expression, leading to hypermethylation and impaired gene activation, while hypomethylation in regions like the *P73* promoter correlates with oocyte senescence [158], underscoring the dual and context-dependent role of methylation dynamics in ovarian aging

**b. Histone Modifications and Chromatin Remodeling:** Histone modifications, including acetylation, methylation, and phosphorylation, play a central role in regulating chromatin accessibility and gene transcription [171]. In POI, reduced levels of the activating histone marks H3K27ac and H3K4me3 have been observed in lymphoblastoid cell lines derived from patients with X-autosome translocations, indicating a global disruption of chromatin regulation [5]. Notably, these epigenetic alterations extend beyond chromosomal breakpoints, supporting the “position effect” hypothesis whereby chromosomal rearrangements perturb higher-order nuclear architecture and regulatory landscapes [5]. In animal models, *HDAC6* regulates primordial follicle activation through mTOR signaling, with *HDAC6* overexpression extending reproductive lifespan in mice [172]. In contrast, increased H4K12 and H4K16 acetylation in aged oocytes correlates with chromatin relaxation, compromised chromosomal integrity, and meiotic defects, highlighting histone acetylation as a key regulator of higher-order chromatin organization and meiotic fidelity, rather than gene expression alone [158,173,174].

**c. Non-Coding RNAs and Follicular Atresia:** Non-coding RNAs (ncRNAs)are emerging as key regulators of ovarian function and follicular fate [162,175,176]. In POI, dysregulated microRNAs (miRNAs), including miR-23a, miR-146a, and miR-181a, promote granulosa cell apoptosis by targeting anti-apoptotic pathways and DNA repair mechanisms [177]. In addition, long non-coding RNAs (lncRNAs) have been increasingly implicated in POI pathogenesis [176,178,179]. LncRNAs such as *NEAT1*, *HOTAIR*, and *BBOX1-AS1* modulate granulosa cell survival by interacting with miRNAs or directly regulating transcription factors involved in hormone synthesis, cell-cycle control, and apoptotic signaling [158,179]. For instance, *NEAT1* downregulation increases p53-mediated apoptosis in granulosa cells [180], whereas *HOTAIR* overexpression restores Notch-1 signaling and mitigates cell death [181]. These findings highlight the coordinated role of lncRNA-miRNA regulatory networks in follicular atresia.

Beyond miRNAs and lncRNAs, other classes of ncRNAs are emerging as vital regulators of POI [182,183]. PIWI-interacting RNAs (piRNAs) are essential for maintaining germline genome integrity by silencing transposable elements [184] and are implicated in POI pathogenesis [185]. Additionally, small nuclear RNAs (snRNAs) and small nucleolar RNAs (snoRNAs) participate in RNA splicing and modification critical for oocyte maturation [186]. Recent analyses also highlight the role of tRNA-derived small RNAs (tsRNAs) [187] and rRNA-derived small RNAs (rsRNAs) [188] in transmitting metabolic information, suggesting their dysregulation may contribute to POI risk [182,189]

**d. Environmental and Transgenerational Epigenetic Effects:** Environmental pollutants, including BPA, phthalates, and ionizing radiation, induce epigenetic alterations that impair ovarian function by modifying DNA methylation patterns, histone marks, and ncRNA profiles [163]. These changes disrupt steroidogenesis, compromise granulosa cell function, and ultimately reduce ovarian reserve [24,28,42,43,158,190,191]. In parallel, environmental exposures and chemotherapeutic agents further alter miRNA expression, exacerbating granulosa cell loss and accelerating follicular depletion [158,191,192]. Importantly, transgenerational inheritance of environmentally induced epigenetic alterations has been documented, suggesting ancestral exposures can predispose subsequent generations to POI through heritable disruptions of gene regulatory networks critical for ovarian function [158,190,193,194].

### 4.4. Genotype-Phenotype Correlations

Genetic mutations in POI are increasingly recognized to correlate with the clinical presentation of amenorrhea [57,112]. For example, *FSHR* mutations are predominantly associated with primary amenorrhea [195], reflecting early disruption in follicular recruitment. In contrast, mutations in *AIRE*, *BLM*, and *SPIDR* have been found in secondary amenorrhea cases, suggesting a later-onset ovarian dysfunction [7,63]. Other genes, including *HFM1*, *MSH4*, and *POLG*, span both subtypes, indicating variable expressivity and penetrance [57,196]. These genotype-phenotype distinctions offer valuable insights into disease mechanisms and hold promise for personalized diagnostic and therapeutic strategies [2,3,57].

## 5. Racial and Ethnic Susceptibility in POI

Genetic variants associated with POI differ across racial and ethnic groups, yet most populations remain underrepresented in genomic research. This underrepresentation constrains understanding of differential susceptibility and impedes the development of equitable diagnostic, preventative, and therapeutic strategies.

Ethnic differences in ovarian reserve are well documented across the lifespan. For example, Indian women exhibit ovarian reserve markers comparable to those of Spanish women six years older [197], suggesting underlying genetic or environmental influences on ovarian aging trajectories. African women tend to demonstrate higher ovarian reserve during early reproductive years but experience more rapid age-related declines, whereas Asian women typically show slower declines despite lower baseline reserve levels [198,199,200]. Collectively, these findings indicate that both the timing and rate of ovarian aging vary substantially across populations.

Environmental and lifestyle factors also contribute to racial disparities in POI risk. For example, vitamin D deficiency, more prevalent among African American women due to reduced sunlight exposure, and smoking are both associated with diminished ovarian reserve and accelerated follicular loss [201,202]. These exposures may interact with genetic susceptibility to compound the risk of POI in affected populations.

Systemic and structural barriers also significantly influence POI diagnosis and management. High costs of infertility care, limited insurance coverage, cultural stigma, and healthcare access disparities disproportionately affect minority women, shaping illness perception, help-seeking behaviors, and treatment utilization [203,204]. Stigma surrounding infertility is particularly pronounced among Asian American women, especially those of Chinese descent [205,206,207]. Hispanic, Asian, and Black women express significantly greater concern about others discovering their infertility compared to White women [205]. Specifically, Black women report heightened fears of personal failure and disappointing partners [205], while many Latina women view childlessness as a marital failure [208]. These psychosocial stressors can delay diagnosis and hinder treatment engagement, and are further compounded by language, immigration status, and communication barriers that disproportionately affect non-citizens and recent immigrants [209].

Disparities extend into clinical outcomes, particularly with assisted reproductive technologies (ART) [210,211]. Black, Hispanic, and Asian women experience significantly lower live birth rates compared to White women, with Black women showing the poorest outcomes despite comparable embryo quality and treatment protocols [203,204]. These findings underscore systemic inequities in reproductive care delivery and outcomes rather than biological differences alone.

Genetic predispositions contributing to POI also vary by race and ethnicity. Multiple gene variants affecting ovarian reserve and follicular maintenance have been implicated in population-level differences in POI susceptibility [2,112,212]. The Study of Women Across the Nation reported a POI prevalence of 1.1% among women aged 40–55 years overall; prevalence in Chinese and Japanese women (0.5%) was markedly lower than in Caucasian (1.0%), African American (1.4%), and Hispanic women (1.4%), supporting population-specific genetic and epigenetic influences [213].

Further evidence of ethnic variation in ovarian reserve comes from hormonal biomarker studies. A 2009 study by Seifer et al. demonstrated that Anti Müllerian Hormone (AMH) levels were 25.2% lower in Black women and 24.6% lower in Hispanic women compared with White women among HIV infected cohorts [200]. Subsequent studies confirmed persistently lower AMH levels in Black and Hispanic women, even after adjustment for body mass index, smoking status, and age, suggesting intrinsic biological differences in ovarian reserve dynamics [198]. These disparities are further compounded by historical inequities, cultural mistrust of the medical system, and patterns of “stratified re-production” that continue to constrain reproductive autonomy among minority populations [203,204].

Ethnic disparities in androgen metabolism may also contribute to differential POI risk. African American women exhibit higher frequencies of polymorphisms in *SULT2A1*, which impair DHEA metabolism, as well as elevated SHBG levels that reduce bioavailable testosterone [124]. Together, these metabolic differences may influence follicular survival and ovarian aging trajectories. Collectively, these findings underscore the need to refine diagnostic and therapeutic strategies to account for genetic, metabolic, environmental, and sociocultural variability across populations, reinforcing the importance of truly personalized and equitable reproductive care.

## 6. Systemic Conditions and Consequences of POI

Beyond reproductive dysfunction, POI exerts profound effects on systemic health [18,63,214]. These effects arise through two main mechanisms: pleiotropy, where POI is one of the features of a multi-system disorder caused by a single gene mutation [3,63,123], and hypoestrogenism, the prolonged estrogen deficiency resulting from ovarian failure, regardless of its etiology [1,15,18,21,214].

### 6.1. Pleiotropic Associations: POI as a Multi-System Disorder

Many genes implicated in POI function in ubiquitous cellular pathways, leading to syndromic and/or systemic presentations (Table 2).

**a. Cancer Predisposition:** Defects in DNA damage repair pathways are frequently observed in POI due to pathogenic gene variants [64,65] and are well known to predispose affected individuals to malignancies [215,216]. This shared vulnerability is exemplified by several inherited human syndromes. Fanconi anemia, resulting from mutations in *FANC* and *BRCA* genes, and ataxia telangiectasia, caused by *ATM* mutations, are characterized by genomic instability, increased cancer susceptibility, and ovarian failure or infertility [24,67,70,71,112,217]. Similarly, Bloom Syndrome, caused by mutations in *BLM*, features short stature, cancer risk, and female infertility [218]. Animal models further support this association. For example, *BRCA2* knockout mice recapitulate key features of human POI, including accelerated oocyte loss and infertility, underscoring the essential role of DNA repair pathways in maintaining ovarian reserve [69,70]. Clinically, the relationship between POI and cancer appears bidirectional. Women with POI exhibit an increased risk of certain reproductive cancers [219,220] while the risk of POI is reportedly increased in adolescent and young adult cancer survivors, particularly following gonadotoxic therapies [214,221]. Together, these observations highlight the complex interplay between genomic instability, ovarian failure, and cancer susceptibility.

**b. Neurological Links:** Several POI-associated genes intersect with neurological pathways, exemplifying pleiotropy. Perrault Syndrome, an autosomal recessive disorder, combines sensorineural hearing loss with POI and is linked to genes like *CLPP* and *HARS2* [222]. It can also involve progressive neurological symptoms like ataxia [222]. Variants in *POLG*, a key regulator of mitochondrial DNA replication and repair, have been linked to both POI and Parkinson’s disease, reflecting shared vulnerabilities related to mitochondrial dysfunction and accumulated cellular damage [118,150,223]. Similarly, *GPR3* has been implicated in POI and independently associated with Alzheimer’s disease, although the underlying mechanisms may differ between ovarian and neuronal contexts [96,224]. Developmental and metabolic syndromes further illustrate the systemic reach of POI. Blepharophimosis-Ptosis-Epicanthus Inversus Syndrome (BPES), caused by *FOXL2* mutations, combines POI (Type 1 BPES) with distinctive eyelid malformations [114,225]. Galactosemia, due to *GALT* mutations, leads to toxic galactose accumulation and POI, even in treated individuals, alongside neurodevelopmental delays [115,226,227]. Together, these examples underscore the convergence of reproductive and neurological phenotypes through shared genetic pathways and reinforce the concept of POI as part of broader systemic and neurodevelopmental networks.

**c. Autoimmunity:** Autoimmune mechanisms account for up to 20–30% of POI cases, with increased risk in conditions such as Hashimoto thyroiditis, Graves’ disease, and type 1 and type 2 diabetes [3,47]. Mutations in *AIRE* cause autoimmune polyglandular syndrome (APS), presenting with a triad of Addison’s disease, hypoparathyroidism, and POI [228]. Reduced expression of *FOXO3α* has been observed in patients with rheumatoid arthritis, systemic lupus erythematosus, and inflammatory bowel disease [139,229,230]. Additional autoimmune disorders associated with this condition include hypophysitis, idiopathic thrombocytopenic purpura, vitiligo, alopecia, autoimmune hemolytic anemia, pernicious anemia, Sjogren’s syndrome, primary biliary cirrhosis, and chronic active hepatitis [3,13,15,47,62,116].

**Table 2:** Representative Systemic Health Conditions Associated with POI-Related Genes.

Associated Condition	Key POI-Related Genes	Summary of Findings
Cancers	*BRCA2*, *MSH4*, *WT1*	Associated with Breast Cancer. *BRCA2* mutations are a well-established risk factor [231]. Polymorphisms in *MSH4* [232] and *WT1* [233] are also linked.
	*DMC1*, *RECQL*, *BRCA*	Associated with Ovarian Cancer [234]. *BRCA* mutations increase the risk of serous ovarian cancer [235].
	*MCM9*, *LHX8*, *POLG*	Implicated in Cervical Cancer progression, especially in HPV-positive cases [236,237,238].
	*MCM8*, *POLG*	Overexpression of MCM8 correlates with poor Gastric Cancer prognosis [239]; *POLG* underexpression is linked to reduced survival [240].
	*BRCA2*, *MCM8*, *WT1*	Mutations have been associated with various forms of Leukemia [57,69,233].
Neurological Complications	*MCM8*, *GPR3*	Linked to Alzheimer’s Disease pathology, especially in estrogen-deficient states [224,241].
	*POLG*	Variants identified in Parkinson’s Disease patients, reflecting its role in mitochondrial dysfunction [118,223].
	*MCM9*	Variants associated with meiosis I errors in maternal oocytes, increasing the risk of Down Syndrome [242].
Autoimmunity	*AIRE*	Mutations contribute to Autoimmune Polyglandular Syndromes (APS) and Addison’s disease [46].
	*FOXO3α*	Decreased expression observed in Rheumatoid Arthritis, Inflammatory Bowel Disease (IBD), and Systemic Lupus Erythematosus (SLE) [229].

### 6.2. Consequences of Hypoestrogenism

Separate from genetic syndromes, the loss of ovarian function itself imposes systemic health risks due to the prolonged absence of estrogen [18,20,243]. These effects can occur in any woman with POI, regardless of the cause. Major health impacts include osteoporosis due to rapid bone loss, increased cardiovascular risk through endothelial dysfunction and adverse lipid changes, and a range of neurological and psychosocial effects (discussed below). These findings underscore the need to view POI not only as a reproductive disorder but as a systemic condition with broad clinical implications requiring long-term multidisciplinary management [3,18].

## 7. Psychosocial Impact of POI

**Figure 2:**
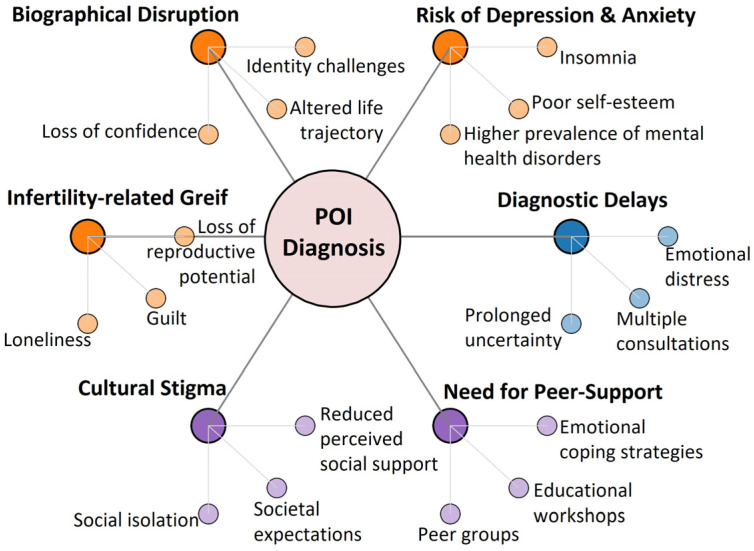
**The Multifaceted Psychosocial Burden of POI.** A concept map illustrating the emotional and social consequences of a POI diagnosis, highlighting the key domains of patient impact and underscoring the need for tailored psychosocial interventions.

POI exerts a profound and multifaceted impact on mental and emotional well-being, extending well beyond reproductive concerns [244,245,246] (Figure 2).

Women with POI frequently report impaired quality of life, often driven by the life-altering diagnosis of infertility [247]. In a cross-sectional study by Singer et al. [248], most participants described the diagnosis as traumatic, a reaction compounded by limited awareness of POI and inadequate guidance from healthcare professionals. As a consequence, many women turned to online resources rather than medical experts for information and support. Younger patients expressed heightened anxiety about physical development and growth, underscoring the strong influence of age and life stage on the psychosocial burden of POI.

Recent meta-analyses and large cohort studies confirm that POI is strongly associated with mental health disorders [244,245,249]. A 2024 systematic review and meta-analysis by Tian et al. [245] found that women with POI have a 2.7-fold higher risk of depression and a 3.7-fold higher risk of anxiety compared to age-matched controls. POI was also linked to a 2.6-fold increased risk of poor quality of life, encompassing fatigue, guilt, grief, loneliness, insomnia, and impaired self-esteem. These findings were consistent across idiopathic and iatrogenic POI subtypes, with unmarried women showing particularly elevated risks for depression [245]. In a 2025 cross-sectional study of 345 women [244], 29.9% reported clinically significant depressive symptoms, with severity strongly associated with younger age at diagnosis, severe menopausal symptoms, lack of emotional support, and fertility-related grief. Notably, estradiol levels and hormone therapy did not correlate with depression [244], emphasizing the primacy of psychosocial over hormonal determinants of mental health outcomes in POI.

Delays in diagnosis further exacerbate emotional distress [250,251]. Alzubaidi et al. [252] reported that 25% of women waited over five years for a confirmed diagnosis, with more than half consulting at least three clinicians before appropriate testing. During this prolonged diagnostic journey, many experienced shock, denial, anger, and grief, reflecting the psychological toll of uncertainty and misdiagnosis.

The diagnosis of POI also affects self-perception, identity, and confidence [245,253,254], with 78% of women reporting long-term emotional consequences [245]. Lifetime depression rates in POI approach 54.5%, often intensified by the intersection of infertility, familial expectations, and societal roles [245]. The concept of *biographical disruption*, a sudden break in life trajectory and identity, emerges as a central theme in POI-related distress [244,255]. Loss of fertility, altered life goals, and perceived social stigma amplify vulnerability to depression and anxiety [253]. Comparable psychosocial patterns are observed in Turner syndrome, where delayed diagnosis is associated with increased odds of depressive symptoms, poor self-image, and higher rates of substance use, underscoring shared mechanisms of chronic reproductive disruption [256].

Cultural norms and socioeconomic pressures further amplify the emotional burden of POI [251,257]. In many societies, reproductive capacity is closely tied to identity and social value, making POI not only a medical condition but also a source of stigma and isolation [257]. Cross-cultural studies confirm that infertility stigma and reduced perceived social support are significant contributors to poor quality of life in POI patients [244]. Importantly, nearly 69% of participants in the Singer et al. study expressed a strong interest in peer support groups and educational workshops [248], emphasizing the unmet need for structured, culturally sensitive psychosocial interventions.

Emerging evidence also suggests a bidirectional relationship between psychosocial stressors and POI risk [39,246,258]. Large-scale meta-analyses indicate that Type A personality traits, characterized by competitiveness, time urgency, and perfectionism, are associated with a six-fold increased risk of POI [38]. Chronic stress, sleep deprivation, and negative mood states were also identified as significant risk factors, with odds ratios ranging from 3.3 to 4.7 [39]. Together, these findings suggest that psychological distress may not only arise as a consequence of POI but may also contribute to its onset, potentially through dysregulation of the hypothalamic-pituitary-ovarian axis and stress-induced epigenetic modifications.

## 8. Discussion and Future Directions

POI research is shifting from single-gene etiologies toward broader epigenetic, environmental, microbial, and lifestyle determinants [39]. Despite these advances, most POI cases remain idiopathic, indicating that current diagnostic frameworks incompletely capture its multifactorial and polygenic nature [62]. As reviewed here, POI frequently arises from complex gene-gene interactions or syndromic involvement, complicating genotype-phenotype correlations and limiting clinical translation [28,196,259]. Advances in high-throughput sequencing, spatial transcriptomics, and CRISPR-Cas9 are redefining ovarian aging biology and enabling earlier detection and more personalized interventions [260].

Host-microbiome interactions represent an emerging frontier in POI research [45,261]. The gut and vaginal microbiome influence ovarian function through effects on estrogen metabolism, immune signaling, oxidative stress, and follicular development [39,45,261]. Dysbiosis may therefore represent a modifiable risk factor, offering opportunities for preventative and adjunctive interventions such as dietary modulation and probiotic therapy.

A major unmet need in POI care is the identification of robust biomarkers that predict ovarian decline before irreversible follicular loss, alongside effective fertility-preserving or restorative strategies [3,28]. The DNA repair-POI axis presents a particularly promising path for biomarker discovery [59,64,65,112]. Regulators of HR, including RAD51 and the meiosis-specific DMC1, maintain oocyte genome integrity and merit systematic biochemical and cellular validation as candidate biomarkers and therapeutic targets [262]. Integrating functional assays with longitudinal clinical cohorts and multi-omics approaches spanning genomics, epigenomics, and microbiomics will be essential to improve diagnostic precision and elucidate disease mechanisms.

Epigenetic profiling also offers opportunities for both risk stratification and intervention. DNA-methylation-based epigenetic clocks may forecast ovarian reserve and reproductive-aging trajectories [161]. Preclinical studies demonstrate that epigenetic therapies, including histone deacetylase inhibitors [38,162], miRNA modulators [263], and stem cell-derived exosomes [27,264], can partially restore ovarian function [28,265]. For example, bone marrow mesenchymal stem cell exosomes carrying miR-144-5p [266] or miR-644-5p [267] reduce granulosa cell apoptosis and improve ovarian outcomes in animal models. Lifestyle interventions such as regular physical activity and vegetable-rich diets may further mitigate POI risk by modulating oxidative stress and hormonal homeostasis [44].

Clinically, translating genetic insights into proactive care is critical. Women carrying pathogenic variants in DNA repair pathways may benefit from earlier fertility preservation, including oocyte cryopreservation before substantial ovarian reserve depletion [18]. Given evidence that mitochondrial dysfunction contributes to ovarian aging and infertility [52,55], and that menopausal hormone therapy may help mitigate aspects of accelerated biological aging [22,268], further mechanistic and clinical studies of these interventions are warranted.

Beyond its immediate clinical relevance, POI provides insight into the physiology of ovarian aging. Large-scale genomic studies identify signals in DNA repair genes that influence both age at natural menopause and ovarian reserve [7,269], reinforcing the central role of genome stability in reproductive lifespan [270]. Understanding these shared mechanisms may inform strategies to extend fertility potential and improve long-term health.

Alongside biological discovery, future research must address persistent gaps in population representation and the interplay between psychological and biological factors. A large proportion of genomic studies have focused on Han Chinese cohorts; inclusion of underrepresented African American and Hispanic populations is essential for clarifying ethnic-specific pathways, such as androgen metabolism and stress responsiveness [124]. Equally important is elucidating the bidirectional relationship between POI and mental health [258,271]. Recent evidence that chronic psychological stress may contribute to POI pathogenesis in some individuals necessitates integrative models linking sustained stress, hypothalamic–pituitary–adrenal axis dysregulation, and epigenetic remodeling to ovarian dysfunction [39].

Integrating biological understanding with equity-focused approaches is central to advancing POI care toward early intervention, precision prevention, and lifelong reproductive health. Priorities include early risk detection, fertility-preserving strategies, inclusive genomic research, and culturally tailored support. Mechanistic insights into genome stability, mitochondrial function, epigenetic remodeling, and lifestyle factors can drive the development of actionable biomarkers and targeted therapies. Leveraging POI as a model of ovarian aging can shift care from late-stage management to proactive, precision interventions that safeguard reproductive potential and extend women’s health span.

## Data Availability

No new data were created or analyzed in this study.
